# Mobgap: A State-of-the-Art Python Framework for Reproducible Estimation and Algorithm Validation of Digital Mobility Outcomes from a Single Wearable Device

**DOI:** 10.3390/s26134294

**Published:** 2026-07-06

**Authors:** Cameron Kirk, Arne Kuederle, Paolo Tasca, Metin Bicer, Dimitrios Megaritis, Eran Gazit, Tecla Bonci, Anisora Ionescu, Chloe Hinchliffe, Alexandru Stihi, Anika Muecke, Zamal Babar, Ioannis Vogiatzis, Bjoern Eskofier, Claudia Mazzà, Andrea Cereatti, Arne Mueller, Daniel Rooks, Brian Caulfield, Lynn Rochester, Silvia Del Din

**Affiliations:** 1 Translational and Clinical Research Institute, Faculty of Medical Sciences, Newcastle University, Newcastle upon Tyne NE4 5PL, UK; cameron.kirk@newcastle.ac.uk (C.K.); metin.bicer@newcastle.ac.uk (M.B.);; 2 Department Artificial Intelligence in Biomedical Engineering, Friedrich-Alexander-Universität Erlangen-Nürnberg, 91054 Erlangen, Germany; a.kuederle@gmail.com (A.K.);; 3 Department of Electronics and Telecommunications, Politecnico di Torino, 10129 Turin, Italy; paolo.tasca@polito.it (P.T.);; 4 Department of Sport, Exercise and Rehabilitation, Northumbria University, Newcastle Upon Tyne NE1 8ST, UK; d.megaritis@northumbria.ac.uk (D.M.);; 5 Center for the Study of Movement, Cognition and Mobility, Neurological Institute, Tel Aviv Sourasky Medical Center, Tel Aviv 64239, Israel; erang@tlvmc.gov.il; 6 School of Mechanical, Aerospace and Civil Engineering, The University of Sheffield, Sheffield S10 2TN, UK; 7 Laboratory of Movement Analysis and Measurement, Ecole Polytechnique Federale de Lausanne, 1015 Lausanne, Switzerland; 8 Department of Mechanical Engineering, Insigneo Institute for in Silico Medicine, The University of Sheffield, Sheffield S10 2TN, UK; 9 Ludwig-Maximilians-Universität München, 80539 Munich, Germany; 10 Institute of AI for Health, German Research Center for Environmental Health, Helmholtz Zentrum München, 85764 Neuherberg, Germany; 11 Indivi AG, 4051 Basel, Switzerland; 12 Translational Medicine, Biomedical Research, Novartis, 4056 Basel, Switzerlanddaniel.rooks@novartis.com (D.R.); 13 School of Public Health, Physiotherapy & Population Science, University College Dublin, D04 V1W8 Dublin, Ireland; 14 Insight Research Ireland Centre for Data Analytics, University College Dublin, D04 V1W8 Dublin, Ireland; 15 National Institute for Health and Care Research (NIHR), Newcastle Biomedical Research Centre (BRC), Newcastle upon Tyne NE4 5PL, UK; 16 Newcastle upon Tyne Hospitals, NHS Foundations Trust, Newcastle upon Tyne NE7 7DN, UK

**Keywords:** digital health, wearables, open source software, gait analysis, Python, real world

## Abstract

Objective, continuous assessment of real-world mobility using wearables has significant potential to transform clinical research and practice, yet the field lacks standardised, open-source tools that enable reproducible algorithm real-world validation, across multiple clinical cohorts. This would improve transparency around definitions and performance, thereby enhancing interpretation and more meaningful comparison across studies. The Mobilise-D consortium validated a comprehensive analytical pipeline for estimating digital mobility outcomes from wearables, originally implemented in a combination of MATLAB, R, and Python codes. To overcome the licencing, reproducibility, and accessibility limitations of this implementation, the pipeline has been re-implemented and re-validated, against gold standards, as the open-source mobgap Python package. Here, we describe the mobgap ecosystem, detail how algorithms can be integrated and benchmarked in a reproducible way and present a re-validation of the pipeline against reference data across six clinical cohorts under real-world conditions. Validation results showed that across all cohorts, walking speed was estimated with an absolute error of 0.10 m/s and an intraclass correlation coefficient (ICC) of 0.81, demonstrating comparable or superior performance to the original implementation. Mobgap (v1.2) is openly available and is intended to serve as a reproducible reference implementation and benchmarking platform for researchers developing or validating mobility analysis algorithms using wearable data.

## 1. Introduction

Despite growing clinical interest, mobility assessment in intervention trials remains largely reliant on self-report or brief, clinic-based tests that capture mobility capacity rather than real-world mobility performance, thereby limiting ecological validity and therapeutic insight [1,2,3,4]. Wearable digital health technologies enable objective, continuous assessment of real-world mobility performance through digital mobility outcomes (DMOs) [5], with walking speed being the most widely studied due to its strong associations with ageing, disease, falls, and mortality, as well as its clinical interpretability [4]. However, there is an overreliance on proprietary and unvalidated software and algorithms used to estimate DMOs in large clinical trials in non-academic and academic settings. Selective DMOs have recently undergone extensive validation through the multi-centre Mobilise-D research project [5,6], which developed and rigorously validated an analytical pipeline for DMO estimation, achieving state-of-the-art accuracy and reliability across laboratory and real-world conditions and across multiple clinical cohorts [7,8,9,10]. The Mobilise-D pipeline comprises a sequence of analytical blocks, including gait sequence detection, initial contact detection, stride length estimation, cadence estimation, and walking speed estimation [11]. For each block, candidate algorithms were identified from the literature and systematically ranked based on their validity [7]. These algorithms were originally implemented as the ‘Mobilise-D pipeline v1.0’, using a combination of MATLAB, the most widely used academic programming language, and Python, requiring Docker containers [12] to ensure execution within a unified workflow. However, this approach increased processing times and MATLAB requires a paid licence and is subject to software versioning that may compromise long-term reproducibility, constraining the pipeline’s potential for open and sustainable dissemination. Such limitations do not apply to Python-based implementations. While open-source Python tools exist, such as GaitPy [13], SciKit Digital Health [14] and gaitmap [15], these packages have not been validated in real-world settings, or in the case of gaitmap utilise different multi-sensor wearable device configurations.

Beyond the specific limitations of the original implementation, a broader challenge exists in comparing the performance of different algorithms on common, publicly available datasets. Without a dedicated validation framework, such comparisons are not performed routinely, limiting both the direct assessment of validity across studies and the adoption of new techniques. Additionally, the technical complexity of implementing validation analyses in a fully reproducible way, ensuring that identical statistical procedures and performance metrics are applied consistently across studies and datasets, represents a substantial barrier. Without standardised, end-to-end tooling, replication and fair algorithmic comparison remain difficult in practice, impacting the speed at which improvements or additions to these software pipelines can take place. The present work therefore goes beyond a straightforward translation from MATLAB to Python, introducing a structured framework for validating each block of the processing pipeline, enabling any new or existing algorithm to be assessed in a metrics-driven and fully documented fashion against common, publicly available datasets.

To address both challenges, the Mobilise-D pipeline was re-implemented within the open-source mobgap Python package, available on GitHub (v1.2) under an open-source Apache 2.0 licence [16], resolving the licencing and reproducibility limitations of the original implementation. Additionally, the original pipeline was not published in full, meaning mobgap is the true published implementation. Mobgap provides end-to-end framework designed to enable researchers to validate any set of algorithms on any standardised dataset in a fully reproducible way [17]. To demonstrate this capability, we present a comprehensive revalidation of the mobgap pipeline on the Mobilise-D Technical Validation Study (TVS) dataset [18], the largest open validation dataset for inertial measurement unit (IMU)-based gait analysis, both at the level of individual algorithmic blocks and for the full pipeline. This revalidation simultaneously serves as a direct comparison between the Mobilise-D pipeline v1.0 and the new mobgap pipeline, establishing a reproducible baseline against which future algorithmic improvements can be benchmarked. Adherence to the V3+ framework [5,19,20,21] ensures that sensor-derived DMOs generated by mobgap meet the standards required for use as endpoints in interventional trials and regulatory submissions, addressing a critical gap between the scientific validation of wearable sensor algorithms and their practical adoption in research and clinical settings.

This technical note presents mobgap, an open-source Python library for estimation of DMOs from a single lower-back-worn device, and provides guidance on extending, benchmarking, and reporting results from the pipeline.

There are four objectives:

(i) Describe the mobgap library ecosystem, including the data formats and structures required to run the pipeline and how to interpret its outputs;
(ii) Detail how new algorithms can be integrated into the pipeline and benchmarked against existing implementations in a reproducible way, lowering the barrier for researchers seeking to validate or compare novel approaches on standardised datasets;
(iii) Validate the performance of the mobgap pipeline against a gold-standard reference system across multiple clinical cohorts under real-world conditions and compare to the original Mobilise-D MATLAB implementation, establishing the largest reproducible benchmark and performance baseline for lower-back-worn IMUs;
(iv) Provide practical guidance on how to use and report mobgap outputs appropriately in research and clinical contexts, including recommended reporting precision for aggregated DMOs.

## 2. Materials and Methods

### 2.1. Validation Dataset

The mobgap pipeline has been validated in its performance upon the participants that were recruited as part of the Mobilise-D TVS, with ethical approval granted by relevant ethical committee at each of the study sites. Complete ethical statements, protocol, recruitment and inclusion criteria are described in more detail in the literature [6,7,8,9], and the TVS dataset is now publicly available on Zenodo [18].

Participants were recruited from five sites throughout the United Kingdom, Germany and Israel among people with congestive heart failure (CHF), chronic obstructive pulmonary disease (COPD), multiple sclerosis (MS), Parkinson’s disease (PD), proximal femoral fracture (PFF) and healthy age-matched controls (HA). Informed consent was provided by all participants to take part in the study, and all research was performed in accordance with the Declaration of Helsinki. Participants were assessed in the laboratory and for 2.5 h in the real world. They wore a McRoberts Dynaport 6 MM+ inertial device (McRoberts, The Hague, Netherlands) with a belt (tri-axial accelerometer range of ±8 g, tri-axial gyroscope range: ±2000 degrees per second (dps), sampling frequency 100 Hz). Participants also wore the multi-sensor INDIP system (sampling frequency: 100 Hz), which served as the validation reference system [6,10,22].

### 2.2. Overview over the Mobgap Ecosystem

**Figure 1:**
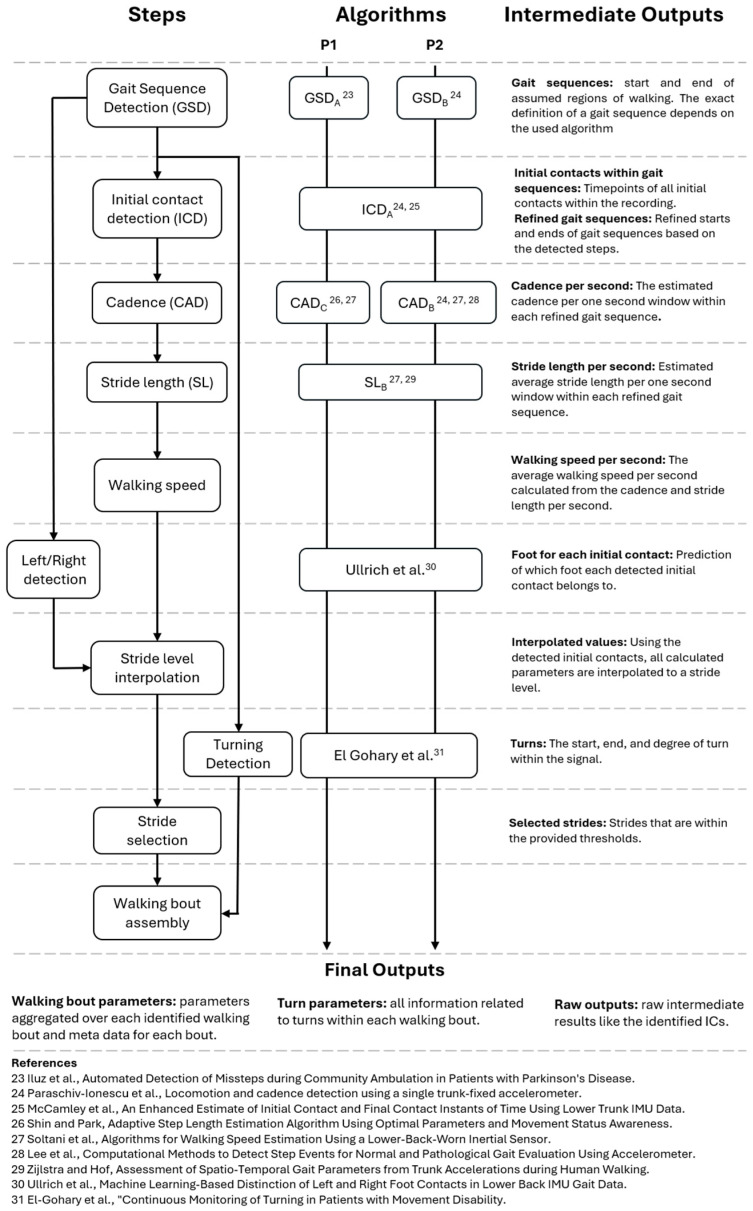
Overview of the mobgap analytical pipelines P1 optimised for non-impaired walkers and P2 optimised for impaired walking. Reproduced from Kirk et al., Scientific Reports, 2024, 14, 1754, under a Creative Commons Attribution 4.0 International Licence (CC BY 4.0): https://creativecommons.org/licenses/by/4.0/, accessed on 14 May 2026. IC = Initial contact. For complete details of the pipeline see [7,9]. References for each included algorithm as follows: refs. [23,24,25,26,27,28,29,30,31].

The mobgap package includes all required algorithms for estimation of DMOs from a wearable device utilising an analytical pipeline that was validated by the Mobilise-D consortium [7,9]. Mobgap provides algorithms for each block of the pipeline, and an overview of the pipeline and the respective references are provided in Figure 1. Mobgap provides two validated pipelines optimised for individuals with non-impaired (P1) and impaired walking (P2). The only difference between the P1 and P2 is different algorithms for GSD and cadence estimation (Figure 1).

Each algorithm is implemented as a Python class, providing attributes to configure the algorithm parameters. The algorithmic blocks can be applied independently or combined into a pipeline for end-to-end processing. For each step we provide algorithm implementations that can be used interchangeably in a pipeline or can be completely substituted by custom implementations. Beyond the core algorithms, the package provides methods to preprocess data, simplify the development of custom algorithms, and to evaluate the output of algorithms against ground truth references.

### 2.3. Data Standardisation

To enable reproducible algorithm development and fair comparison across datasets and studies, mobgap defines standardised input and output datatypes with explicit conventions for units, coordinate systems, and data structures. These conventions are designed to ensure consistency across pipelines while remaining flexible to accommodate different sensor placements and study designs. Mobgap follows the International System of Units (SI) wherever possible for all physical quantities. Acceleration is expressed in metres per second squared (m·s^−2^), angular velocity in degrees per second (dps), temporal quantities in seconds (s), and spatial measures such as stride or step length in metres (m). Angular quantities are represented in degrees rather than radians to improve interpretability for clinical and biomechanical applications. Mobgap assumes uniformly sampled data; all internal event representations use sample indices, with time derived implicitly from the known sampling rate, and absolute timestamps are not required as algorithmic input. Recording start time, where available, is stored as metadata in ISO 8601 [32] format with time zone information (e.g., 2023-10-26T15:30:00+05:00).

**Figure 2:**
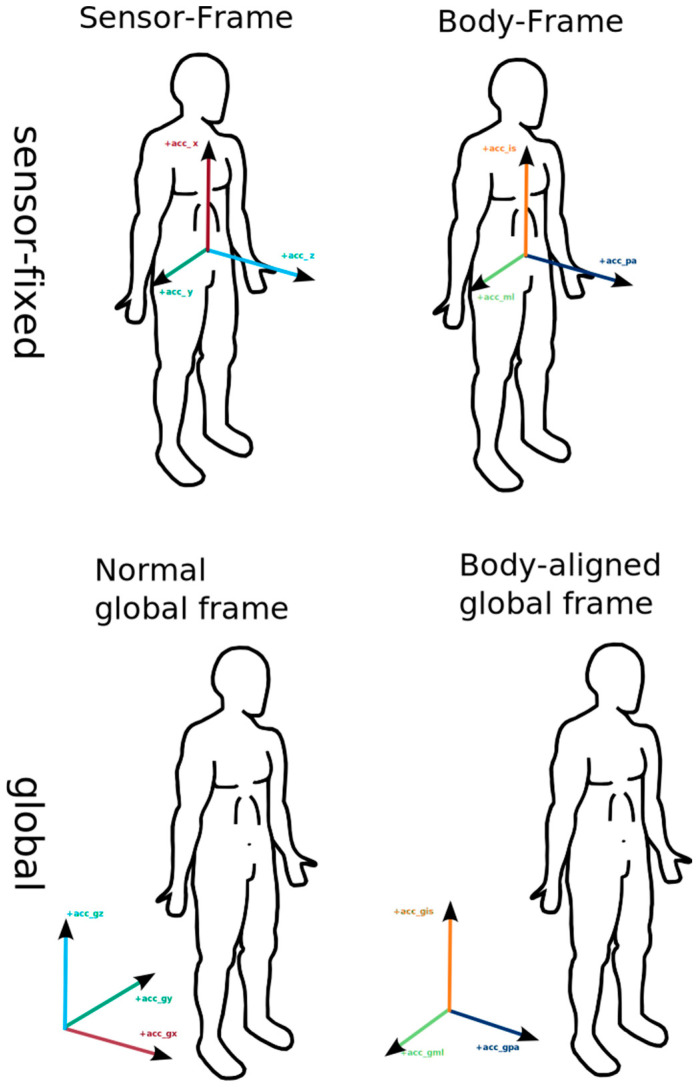
Reference figure for different coordinate systems. Legend for subscripts: g = global; is = inferior–superior; pa = anterior–posterior; ml = medial–lateral.

The choice of coordinate system depends on the processing stage and IMU location. For lower-back-worn IMUs, Mobgap distinguishes between two coordinate systems (Figure 2):

- Sensor coordinate system
  ○ The unit coordinate system is defined by the physical axes of the IMU sensors aligned with the unit IMU casing. By convention, the x-axis points upward relative to the participant’s body, the y-axis to the right and the z-axis points approximately anteriorly. Raw accelerometer and gyroscope signals are provided in this frame.
- World (global) system
  ○ The world system has the vertical axis coinciding with the gravity direction and is used to describe global orientation, such as estimating walking direction, vertical displacement, or accumulated trajectory.

All signals and derived outputs in mobgap are represented using standardised tabular data structures, typically pandas DataFrames [33] with a time-based index. Columns correspond to well-defined physical quantities (e.g., acceleration, angular velocity, orientation), with consistent naming conventions and documented units. Higher-level datatypes, such as stride lists, event annotations, or gait parameter tables, build upon these basic structures and enforce explicit relationships between time, sensor data, and derived measures. By standardising units, coordinate systems, and data representations, mobgap ensures that algorithms can be applied consistently across datasets, reduces ambiguity in interpretation, and supports transparent benchmarking of gait analysis inertial-based methods in biomedical research and regulatory qualification [34].

### 2.4. Extending Mobgap

Each algorithmic block in mobgap is implemented as a Python class that adheres to a clean interface separating parameters to configure an algorithm and the actual execution of the algorithms. A minimal implementation of an algorithm can be seen in Supplementary Text Box S1. This approach inspired by *sklearn* [14], and previously implemented in *gaitmap* [15] via the *tpcp* [35] library, enables the substitution of one algorithm implementation for another using dependency injection [15].

Algorithms do not have to be actively added to mobgap to be usable as part of it. When using mobgap as a library, during the definition of a pipeline object any instance of a Python class adhering to the defined interface can be used for each algorithmic block. Alternative algorithm implementations can either be developed by the user or provided from other libraries that provide mobgap compatible implementations (Supplementary Text Box S1). For trainable algorithms such as those based on machine learning (e.g., Left–Right detection and stride length estimation), mobgap’s evaluation framework supports train–test splits and k-fold cross-validation via the EvaluationCV class, ensuring that optimisation and evaluation are cleanly separated across data folds.

This allows full flexibility and the use of local proprietary algorithms in combination with the provided open-source pipeline, enabling anybody to propose new algorithms, without being dependent on mobgap’s development cycle. At the same time, we are planning to expand the list of algorithms directly available in mobgap through continued development funded by research institutes that were part of Mobilise-D and community contributions.

### 2.5. Benchmarking

Revalidation of any new algorithm must be evaluated considering performance of the individual algorithm block, in comparison to the existing mobgap block and to the reference data, and validation of walking speed estimation to determine whether the new block influences performance of the full pipeline. The core blocks of the pipeline include gait sequence detection, initial contact detection, stride length estimation, cadence estimation and walking speed estimation. Additional blocks such as laterality (left–right: LR) detection or turning can also be re-evaluated. Validation must be determined independently for each cohort by comparing DMOs obtained from the new algorithm to mobgap and those estimated from the reference systems. As per the Mobilise-D guidelines, the new method must be evaluated within its specified context of use, such as type of assessment (real-world or lab), and specific patient cohorts [21].

The validation approach, performance metrics and statistical analysis have been previously published, comparing each algorithmic block of the pipeline (Figure 1) [7], along with the full pipeline performance in its estimation of walking speed in comparison to the reference system [9].

Mean and 95% confidence intervals of all DMOs are evaluated at a cohort level (i.e., quantified using all trials across all participants belonging to that specific cohort). For estimation of stride length, cadence and walking speed subsets of relevant measures are used for the different digital mobility outcomes and evaluated as detailed below.

### 2.6. Analysis Examples

In this technical note, we have included a few analysis examples based upon the real-world data from the TVS dataset to provide a high-level summary of the full pipeline performance, selected block-by-block validation and of how to report the outputs of the mobgap pipeline within a clinical study. The full validation results of the mobgap pipeline have been published in greater detail within the mobgap documentation [16]. We also report mobgap and the original Mobilise-D v1.0 performances compared separately against the reference system for the block-by-block and full pipeline approaches.

#### 2.6.1. Example #1. Block-by-Block Evaluation Approach

Performance of the full pipeline can be influenced by the accumulation of errors from the preceding blocks. Therefore, blocks must be evaluated independently alongside estimates of the full pipeline to quantify the amount of error introduced by each block. In this case the input data is represented by the individual output of the reference system output of the corresponding block, as described previously [7], with the validation to be repeated in the lab and the real-world validation comprising the 2.5 h assessment. Here, we provide an example performance of the core mobgap algorithms in relation to the reference system, utilising selected statistical output from the benchmarking approach, for only the 2.5 h real-world validation. The examples we provided are only those from all walking bouts (WBs) that are matched between the reference system and mobgap; for more information see [7]. Performance of each algorithmic block is additionally compared directly to the corresponding original Mobilise-D pipeline v1.0 implementation using paired Wilcoxon signed-rank tests with Benjamini–Hochberg false discovery rate correction applied across all cohort-level comparisons. The scripts used to generate all figures and perform all statistical comparisons are available in the mobgap GitHub to ensure full reproducibility of the results presented here [https://github.com/mobilise-d/mobgap/tree/main/scripts (accessed on 20 January 2026)].

#### 2.6.2. Example #2. Full Pipeline Validation

To provide a representative performance of the combined mobgap pipeline, estimates of walking speed can be compared to the reference system. We applied the mobgap pipelines optimised for impaired and healthy walkers to the relevant cohorts over the 2.5 h real-world assessment undertaken by each of the participants. Utilising the benchmarking approaches outlined in this manuscript, we computed the absolute relative errors, and relative errors alongside bias to show the performance of mobgap compared to the reference system. Pipeline performance was additionally compared directly to the original Mobilise-D pipeline v1.0 using the same method described in Example 1 above.

#### 2.6.3. Example #3. Reporting of Results/Aggregations

We also include results from the mobgap aggregator based upon the clinical validation study dataset [36], which will demonstrate the specific DMOs and their respective aggregations that can be reported from the outputs of mobgap.

Additionally, an important step in reporting the results of mobgap lies in deciding the number of decimal places to report the measurement to, which reflects the precision of the analysis [37]. The golden rule is to always report one digit more than the smallest scale division (resolution). This last digit is the uncertain digit. In the final reported measurement, the last digit is the uncertain one. All previous ones must be certain.

The number of decimal places used to report each aggregated DMO should reflect the effective measurement resolution of the pipeline. Formally, measurement error for observation i is defined as ε_i_ = x_mi_ − x_vi_, where x_m,i_ is the measured value and x_v,i_ is the reference value. Assuming approximately Gaussian-distributed errors, the 95% error range is: mean error ± 2 × stdε. Values are reported to d decimal places, corresponding to one decimal place finer than the measurement resolution implied by σ_e_, with an associated rounding uncertainty of ± 0.5 × 10^−d^.

## 3. Results

### 3.1. Example #1. Block-by-Block Evaluation Approach 

**Figure 3:**
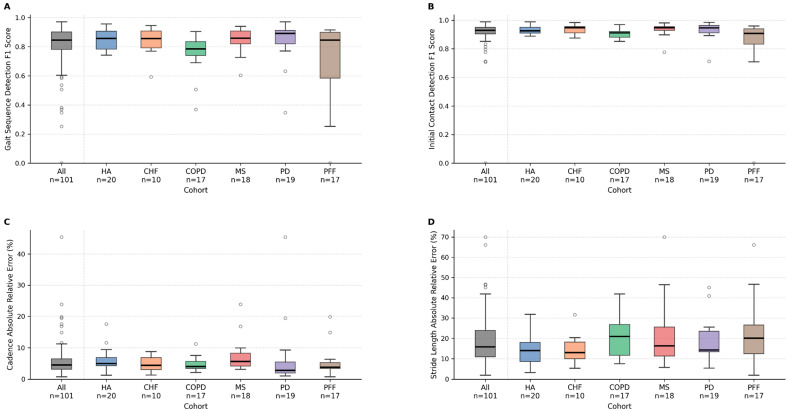
Block-by-block validation performance across clinical cohorts during the free-living 2.5 h assessment. F1 score is shown for gait sequence detection (**A**) and initial contact detection (**B**); absolute relative error is shown for cadence estimation (**C**) and stride length estimation (**D**). HA, healthy adults; CHF, chronic heart failure; COPD, chronic obstructive pulmonary disease; MS, multiple sclerosis; PD, Parkinson’s disease; PFF, proximal femoral fracture. Circles denote outliers, defined as values beyond 1.5 × the interquartile range from the box edges.

Across all cohorts, gait sequence detection achieved F1 scores ranging from 0.72 (PFF) to 0.85 (HA and MS), and initial contact detection achieved F1 scores between 0.84 (PFF) and 0.94 (COPD) (Figure 3A–B). Cadence absolute relative error ranged from 4.75% (COPD) to 7.23% (PFF), with PD showing the weakest agreement (ICC 0.61) compared to ICCs exceeding 0.8 in all other cohorts (Figure 3C). Stride length estimation was the most challenging algorithmic block, with absolute relative errors ranging from 14.26% (HA) to 22.62 (PFF) (Figure 3D; Supplementary Table S1).

**Figure 4:**
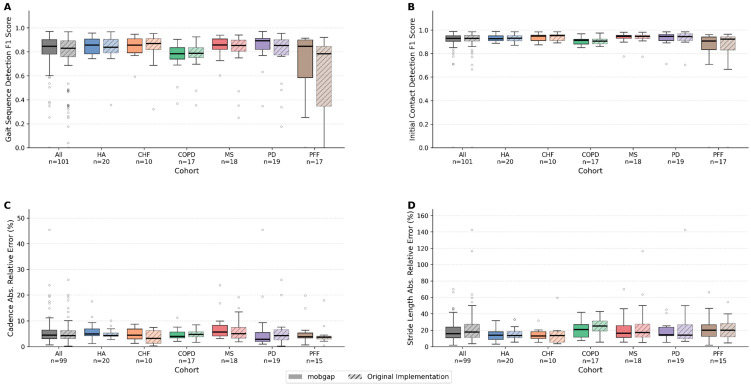
Block-by-block validation performance for mobgap in comparison to the original implementation during the free-living 2.5 h assessment. F1 score is shown for gait sequence detection (**A**) and initial contact detection (**B**); absolute relative error is shown for cadence estimation (**C**) and stride length estimation (**D**). HA, healthy adults; CHF, chronic heart failure; COPD, chronic obstructive pulmonary disease; MS, multiple sclerosis; PD, Parkinson’s disease; PFF, proximal femoral fracture. Circles denote outliers, defined as values beyond 1.5 × the interquartile range from the box edges.

For each cohort, mobgap performance was comparable across each algorithm block, with no significant differences between F1 scores or absolute relative errors after correcting for multiple comparisons (Figure 4). Improvements were consistent across cohorts, with notable ICC improvements in HA (0.88 vs. 0.73), COPD (0.52 vs. 0.36), and PD (0.60 vs. 0.35) (Supplementary Table S2).

### 3.2. Example #2. Full Pipeline Validation

**Figure 5:**
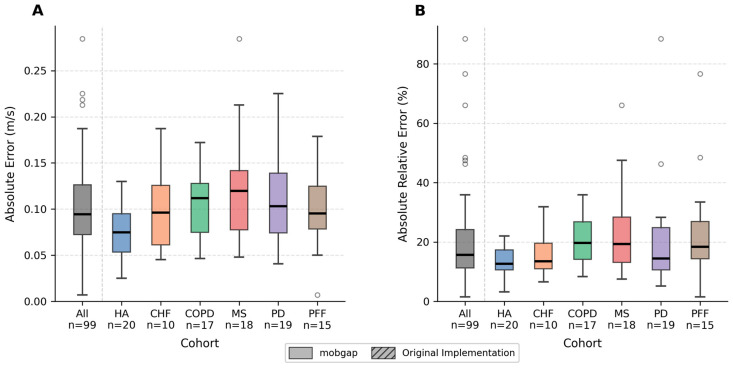
Full pipeline walking speed estimation performance during the free-living 2.5 h assessment. Absolute error (**A**) and absolute relative error (**B**) are shown for walking speed estimated by the mobgap pipeline compared to the INDIP reference system. HA, healthy adults; CHF, chronic heart failure; COPD, chronic obstructive pulmonary disease; MS, multiple sclerosis; PD, Parkinson’s disease; PFF, proximal femoral fracture. Circles denote outliers, defined as values beyond 1.5 × the interquartile range from the box edges.

Across all cohorts, a total of 1984 matched WBs were analysed, ranging from 220 (CHF) to 410 (COPD). Walking speed was estimated with an absolute error of 0.10 m/s and absolute relative error of 19.63%, with a small negative bias of 0.04 m/s indicating a slight tendency to underestimate walking speed (Figure 5). At the cohort level, absolute errors ranged from 0.08 m/s (HA) to 0.12 m/s (MS), with ICC values ranging from moderate to excellent across cohorts (0.53 for COPD to 0.93 for HA) (Supplementary Table S3).

**Figure 6:**
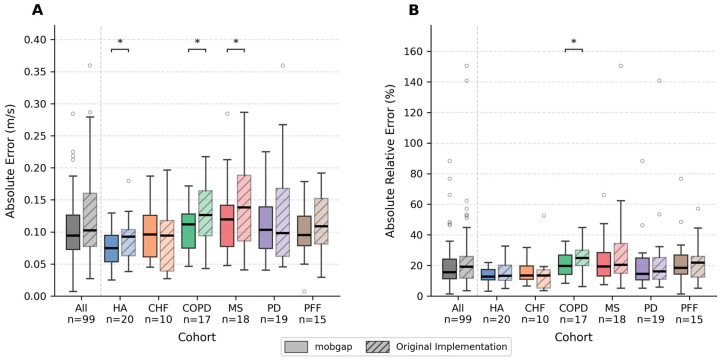
Full pipeline walking speed estimation performance of the mobgap pipeline vs the original implementation across clinical cohorts during the free-living 2.5 h assessment. Absolute error (**A**) and absolute relative error (**B**) are shown for walking speed estimated by the mobgap pipeline compared to the INDIP reference system. HA, healthy adults; CHF, chronic heart failure; COPD, chronic obstructive pulmonary disease; MS, multiple sclerosis; PD, Parkinson’s disease; PFF, proximal femoral fracture. ‘*’ indicates *p* < 0.05. Circles denote outliers, defined as values beyond 1.5 × the interquartile range from the box edges.

Compared to the original pipeline, mobgap had comparable or marginally improved errors for walking speed estimation (Figure 6). Absolute errors were significantly lower for the HA (*p* = 0.029), COPD(*p* = 0.010), and MS (*p* = 0.030), cohorts for mobgap (*p* < 0.05), with significantly lower absolute relative errors for the COPD cohort. Improvements were consistent across individual cohorts, with notable ICC gains in COPD (0.53 vs. 0.40), PD (0.72 vs. 0.53), and PFF (0.79 vs. 0.61) (Supplementary Table S4).

### 3.3. Example #3. Aggregation of Mobgap Outputs

Following a successful pipeline run, results can be summarised at three levels: per stride, per-walking WB, and across the entire recording.

#### 3.3.1. Stride-Level Output

At the stride level, outputs include stride duration, cadence, walking speed, and stride length, alongside the IC timing of each stride and the WB it belongs to. Stride-level outputs are primarily intended for technical validation purposes, where developers can compare the validity of a newly proposed algorithm at this granular level. For clinical reporting, WB-level and recording-level aggregates are recommended instead.

#### 3.3.2. Walking Bout Assembly

Strides are the input to the WB assembly step. WBs are constructed according to a fixed set of consensus definitions established by the Mobilise-D consortium [38] using two sequential components: first, the stride list is filtered using “*StrideSelection*”, which removes strides that do not meet defined quality criteria [7,39]; second, the remaining strides are grouped into WBs using “*WbAssembly*”, which iterates through the stride list and terminates a WB when a stopping criterion is reached (e.g., a pause in walking). Criteria for inclusion of a stride was a duration of 0.2–3 s, with a minimum length of 0.15 m. A resting period or break of ≥3 s identified consecutive WBs; thus, each WB could include resting periods/breaks ≤3 s. An example of the per-WB output produced at this stage is shown in Table 1, including core gait outcomes and the total number of strides per bout.

#### 3.3.3. Recording-Level Aggregation

The full set of WBs across the measurement period is passed as a pandas DataFrame to the “*MobilisedAggregator*”, which aggregates outcomes at the recording level using Mobilise-D consensus thresholds (Table 2). Output variables follow the naming convention wb_[filter]__[metric]__[aggregation], where filter denotes the WB duration threshold (e.g., wb_30 for WBs ≥30 s, wb_all for no filter), metric is the gait parameter, and aggregation is the summary statistic (e.g., avg, p90, var).

### 3.4. Example 4. Recommended Reporting Precision for Aggregated DMOs

Table 3 provides the recommended decimal precision for each aggregated DMO, derived from the error ranges in Supplementary Tables S1 and S2. For example, walking speed is reported to one decimal place (e.g., 0.8 m/s ± 0.05 m/s), as the pipeline absolute error is approximately 0.10 m/s. These recommendations support consistent and appropriately cautious reporting across clinical and research applications.

## 4. Discussion

The applications of DMOs quantified from wearable devices are rapidly growing across academic, industrial, and clinical settings. Until now, a key limitation has been the absence of standardised, open-source tooling to estimate DMOs in a reproducible and accessible way. This technical note presents mobgap, an open-source Python package that provides a straightforward approach to preprocessing, estimating, and summarising DMOs from a wearable device, making the state-of-the-art Mobilise-D pipeline publicly available for the first time as a fully Python-based implementation. Users can now input their own wearable data directly into the pipeline, enabling state-of-the-art DMO processing as a remote monitoring tool within clinical trials, observational studies, and internationally conducted research.

Mobgap represents the most comprehensively validated open-source tool for the quantification of walking and gait characteristics from a lower-back-worn device. Previously published open-source Python packages such as GaitPy [13], SKDH [14], gaitmap [15], and KielIMAT [40] exist; a comparative overview of comparisons across these modular frameworks for extracting clinical features of gait from wearables to mobgap is presented in Table 4. While GaitPy was among the first open-source Python tools for this purpose, its validation evidence across real-world settings and clinical populations was limited in scope compared to mobgap. GaitPy is no longer actively maintained, with its developers recommending migration to SciKit Digital Health (SKDH), a broader Python package that incorporates an updated version of the GaitPy gait module alongside algorithms for sit-to-stand, physical activity, and sleep. SKDH’s gait module showed improved agreement and tighter ICC ranges compared to the original GaitPy implementation in internal validation, though these results were not published in full, limiting independent assessment of its performance across clinical populations and real-world conditions. Importantly, mobgap’s modular, interface-driven architecture means that SKDH-compatible algorithm implementations could in principle be integrated into a mobgap pipeline without modification to the framework itself, offering a potential route to combine SKDH’s broader algorithm library with mobgap’s structured validation and benchmarking infrastructure. The gaitmap package [15] served as an architectural basis for mobgap; however, it utilises foot-worn IMUs and is therefore not directly comparable. In contrast, mobgap has been extensively and openly validated on the Mobilise-D TVS dataset, across six clinical cohorts under real-world conditions, with full results publicly available [7,9,16]. Most fundamentally, mobgap is the only open-source mobility analysis pipeline explicitly aligned to the V3+ regulatory validation framework [5,19,20,21], which defines the statistical requirements and context-of-use criteria for DMOs intended for use as endpoints in clinical trials. This alignment means that mobgap outputs and validation results are directly interpretable within a regulatory context, supporting its use as a clinical outcome assessment (COA) tool.

The revalidation of the mobgap pipeline establishes a concrete, reproducible baseline against which any new method can be benchmarked using the standardised statistical framework presented here. Mobgap demonstrated similar or improved performance relative to the reference system compared to Mobilise-D v1.0 across most metrics. The larger number of WBs detected by mobgap reflects numerical corrections and parameter refinements made during re-implementation of the GSD algorithm, including correction of a signal windowing check. These changes were not intended to alter the fundamental behaviour of the algorithm but collectively resulted in more consistent WB detection. The downstream effect on stride length estimation is consistent with this, as a larger and potentially more representative set of WBs provides a broader basis for estimation. Additional optimisation to stride length estimation, including introduction of a maximum interpolation gap and adoption of linear rather than mean interpolation, further improved robustness to outliers. Mobilise-D v1.0 remains a valid and well-evidenced implementation; these refinements reflect the natural evolution of a re-implemented pipeline rather than correction of fundamental errors.

A key strength of mobgap is its extensibility and support for reproducible benchmarking. Mobgap is built on the tpcp library [35], which provides a scikit-learn-inspired interface that enforces a clean separation between algorithm parameters and execution, and supports dependency injection, enabling any algorithm implementation adhering to the defined interface to be substituted into the pipeline without modification to the surrounding infrastructure. To support fully reproducible benchmarking, validation results are stored on GitHub and can be accessed via the ValidationResultLoader, meaning any researcher can reproduce the exact validation figures presented here without requiring access to the raw TVS dataset. This infrastructure ensures that future algorithm contributions can be directly and transparently compared against the baseline results established in this technical note.

As the field continues to evolve, users may wish to optimise or implement novel approaches, which can be achieved without modification to the surrounding infrastructure, as described in this technical note. One example is a community contribution implementing a laterality algorithm based on [41], which showed comparable performance relative to the original implementation [30], with the added advantage of not requiring gyroscope data. More broadly, the benchmarking infrastructure provided by mobgap enables comparison of any number of algorithm implementations against a standardised baseline. To demonstrate this, Supplementary Figure S1 presents a comparison of three GSD algorithm implementations available within the mobgap, namely GsdIluz, GsdIonescu, and GsdAdaptiveIonescu, evaluated across all six clinical cohorts on the free-living TVS dataset. This comparison required no additional data collection or bespoke analysis code; results were retrieved directly from the validation repository and visualised using the framework described in this technical note. This represents a strong contribution of the mobgap Python framework, enabling algorithm comparisons to be made on publicly hosted benchmarks. For trainable algorithms such as those based on machine learning, tpcp’s evaluation framework additionally supports k-fold cross-validation, ensuring that optimisation and evaluation are cleanly separated across data folds. Given that stride length estimation is the most challenging and assumption-dependent block in this pipeline [7,36,37], it is anticipated that this aspect of mobgap will receive the most significant attention from the research community, and the benchmarking infrastructure described here is well positioned to support and accelerate that development.

Additionally, mobgap could be extended to other wearable device configurations, such as the wrist, building upon earlier approaches from Mobilise-D [42]. A wear-time detection algorithm is being prepared for integration within mobgap. Its absence currently prevents fully autonomous application in clinical trials where wear adherence cannot be assumed; once incorporated, mobgap will assess daily monitoring assessment (DMA) validity according to Mobilise-D criteria prior to aggregation. Note that this does not affect the performance metrics reported here, as continuous wear was ensured during TVS assessments. Mobgap includes an example that replicates the aggregation completed during the clinical validation study see examples, data aggregation in (https://mobgap.readthedocs.io/, accessed on 20 January 2026) which, while it is not a complete implementation, may act as a starting point.

There are limitations associated with mobgap. At present, mobgap includes only spatiotemporal DMOs; however, a large number of signal-based features, including those derived directly from raw accelerometer or gyroscope signals, are currently being prepared for inclusion, with significant work required to establish their reliability and clinical relevance before they can be integrated. Revalidation and benchmarking of the pipeline is based upon the Mobilise-D TVS dataset, which comprises cohorts with specific clinical impairments assessed under both laboratory and real-world conditions. Users wishing to apply mobgap in a context of use, whether a specific cohort, assessment setting, or geographical population, not represented in the TVS dataset should proceed with caution. In such cases, we would recommend collecting a representative local dataset and, where applicable, retraining the machine learning models implemented in the laterality classification and stride length estimation blocks to better reflect the target population. Stride length in particular was most challenging to mobgap pipeline performance, reflecting a limitation of spatial parameter estimation from a single lower-back device. Stride length is not directly measured and is instead inferred from estimating displacement of the vertical centre of mass through use of the inverted pendulum model [43], with the model also taking into account the height of the individual. Thus, estimates can be noisier due to anthropometric assumptions, errors in vertical displacement and initial contact timing. This motivates stride length to become a specific focus in future algorithm developments. We have demonstrated that the mobgap pipeline produces comparable results whether the user employs an AX6 (Newcastle upon Tyne, UK), McRoberts (The Hague, Netherlands) or INDIP device; it is not yet established whether the same holds for other devices, though where sensor placement and configuration are equivalent there is no reason to expect substantially different performance. Users wishing to validate mobgap in alternative device configurations are encouraged to do so and to contribute findings back to the community.

This technical note presents mobgap, an open-source Python framework for the estimation, validation, and benchmarking of DMOs from a single lower-back-worn inertial measurement unit. The re-implementation of the Mobilise-D pipeline was successfully achieved with comparable or improved performance relative to the original MATLAB implementation across all algorithmic blocks and clinical cohorts. Built on the tpcp library, mobgap enables any algorithm to be integrated and benchmarked against a publicly available set of validation results without requiring access to the raw dataset, representing a meaningful advance beyond software migration towards a standardised, reproducible platform for algorithm development and comparison. Limitations include the scope of the TVS validation dataset; users applying mobgap in clinical populations or assessment settings not represented in the TVS should collect representative local validation data. A wear-time detection algorithm and per-day DMO aggregation are not yet implemented and must be handled independently by the user. Future development will focus on stride length estimation, wear-time correction, and expanded device compatibility. Mobgap is expected to be progressively adopted in observational studies, clinical trials, and regulatory submissions as a validated, open-source tool for DMO estimation, with ongoing community contributions continuing to extend its capabilities.

## Figures and Tables

**Table 1:** WB-level output example. This displays an example of four WBs from a participant, with the core output that is provided by mobgap. Each information type is provided in the exact format that is provided by the mobgap pipeline, to the complete resolution.

visit_type	T1				
participant_id	12345				
measurement_date	01/01/2023				
wb_id	0	1	2	3	4
duration_s	4.74702	5.1315	8.52727	16.24554	6.09907
n_raw_initial_contacts	8	7	12	27	8
cadence_spm	99.82188	101.16429	86.53527	91.49977	93.69895
walking_speed_mps	1.16079	2.57881	1.60044	0.95558	2.3323
stride_length_m	2.51885	1.57243	1.66305	0.88961	1.95969
stride_duration_s	1.58675	1.46537	2.56092	3.14549	2.35295
n_turns	0	0	2	1	0

**Table 2:** Example output of the aggregated parameters across the entire measurement recording.

Variable	Value
participant_id	12345
wb_all__count	2378
total_walking_duration_min	632.05
wb_all__n_raw_initial_contacts__sum	59320
wb_all__n_turns__sum	3012
wb_all__duration_s__avg	8.859
wb_all__duration_s__p90	26.927
wb_all__duration_s__var	2.275
wb_all__cadence_spm__avg	94.673
wb_all__stride_duration_s__avg	2.213
wb_all__cadence_spm__var	0.127
wb_all__stride_duration_s__var	0.261
wb_10_30__count	844
wb_10_30__walking_speed_mps__avg	1.497
wb_10_30__stride_length_m__avg	1.865
wb_10__count	1029
wb_10__walking_speed_mps__p90	2.096
wb_30__count	185
wb_30__walking_speed_mps__avg	1.619
wb_30__stride_length_m__avg	1.975
wb_30__cadence_spm__avg	102.81
wb_30__stride_duration_s__avg	2.101
wb_30__walking_speed_mps__p90	2.128
wb_30__cadence_spm__p90	115.18
wb_30__walking_speed_mps__var	0.241
wb_30__stride_length_m__var	0.252
wb_60__count	62

**Table 3:** Recommended decimal precision for aggregated DMO measures.

	Maximal Decimals	Recommended Decimals
**Walking activity—Amount**		
**Walking duration** (min/day)	3	0
**WB step count** (#/day)	-	0
**Walking activity—Pattern**		
**Number of WBs** (#/day)	-	0
**Number of WBs >10 s** (#/day)	-	0
**Number of WBs >30 s** (#/day)	-	0
**Number of WBs >60 s** (#/day)	-	0
**WB duration** (s)	1	1
**P90 WB duration** (s)	1	1
**WB duration bout-to-bout variability** (%)	-	0
**Gait—Pace**		
**Walking speed in shorter** (>10 s–≤30 s) **WBs** (m/s)	2	2
**Walking speed in longer (>30 s) WBs** (m/s)	2	2
**P90 walking speed in WBs >10 s** (m/s)	2	2
**P90 walking speed in longer (>30 s) WBs** (m/s)	2	2
**Stride length in shorter** (>10 s–≤30 s) **WBs** (cm)	0	0
**Stride length in longer (>30 s) WBs** (cm)	0	0
**Gait—Rhythm**		
**Cadence in all WBs** (steps/min)	1	0
**Cadence in longer (>30 s) WBs** (steps/min)	1	0
**P90 cadence in longer (>30 s) WBs** (steps/min)	1	0
**Stride duration in all WBs** (s)	3	2
**Stride duration in longer** (>30 s) WB (s)	3	2
**Gait—Bout-to-bout variability**		
**Walking speed bout-to-b variability between longer (>30 s) WBs** (%)	-	0
**Stride length bout-to-b variability between longer (>30 s) WBs** (%)	-	0
**Cadence bout-to-b variability** (%)	-	0
**Stride duration bout-to-b variability** (%)	-	0

**Table 4:** Comparison of open-source Python packages for wearable mobility analysis.

Feature	mobgap [16]	SKDH [14]	GaitPy	Gaitmap [15]	KielMAT [40]
Sensor placement	Lower back	Lower back/chest	Lower back	Foot-worn	Lower back/multiple
Language	Python	Python	Python	Python	Python
Maintenance status	Active	Active	Discontinued †	Active	Active
DMOs estimated	Total walking duration, WB count stratified by duration threshold, step count, walking speed, stride length, cadence, stride duration, IC timing, turns; aligned to Mobilise-D consensus definitions via MobilisedAggregator	Gait events, temporal parameters, activity, sleep	Gait events, temporal parameters	Stride parameters, gait events, trajectory	GSD, ICD, sit-to-stand, turns, physical activity
Full pipeline (GSD → walking speed)	Yes	No ‡	No	No (foot-worn context)	No
External validation	Extensive (Mobilise-D TVS; 6 cohorts, real-world and lab)	Limited (internal only, not published in full)	Limited (single cohort, lab-based)	Moderate (multiple foot-worn datasets)	Moderate (Mobilise-D and KeepControl datasets)
Extensibility/algorithm substitution	Yes (dependency injection via tpcp)	Partial (BaseProcess subclassing)	No	Yes (tpcp)	Partial
Reproducible benchmarking framework	Yes (end-to-end, standardised metrics)	No	No	Yes (gaitmap challenges/bench, foot-worn context)	No
ML/trainable algorithm support	Yes (cross-validation via tpcp)	Partial (LightGBM classifier integrated)	No	Yes (tpcp)	Partial
Regulatory alignment (V3+/COA)	Yes (Mobilise-D V3+ framework)	No	No	No	No
Target use case	Clinical trials, real-world DMO estimation, algorithm benchmarking	Research, multi-modal daily-life monitoring	Research, clinical gait characterisation	Research, biomechanical gait analysis (foot-worn)	Research, neurological motion analysis

† GaitPy is no longer maintained; users are directed to SKDH. ‡ SKDH provides individual gait modules but does not implement a full end-to-end DMO pipeline aligned to a validation framework.

## Data Availability

The datasets supporting the examples in this technical note can be found publicly available on Zenodo (https://zenodo.org/records/13899386, accessed 12 October 2024). The mobgap analytical pipeline is publicly available for use on Python via GitHub (https://mobgap.readthedocs.io/en/stable/, accessed 20 January 2026).

## Supplementary Materials

The following supporting information can be downloaded at https://www.mdpi.com/article/10.3390/s26134294/s1, Text Box S1: Example of the minimal code required to run the GsdIluz algorithm on the LabExampleDataset evaluation; Table S1: Selected performance measures evaluating the performance of the block-by-block performance measures; Table S2: Selected performance measures evaluating the performance of the block-by-block performance measures of the mobgap pipeline in comparison to the original implementation; Table S3: Full pipeline validation across 2.5-h real-world validation for walking speed; Table S4: Full pipeline validation across 2.5-h real-world validation of the mobgap pipeline in comparison to the original implementation; Figure S1: Example of f1 scores compared across three gait sequence detection (GSD) algorithms that are available within the mobgap repository.
