# Supplementary material for: Mobgap: A State-of-the-Art Python Framework for Reproducible Estimation and Algorithm Validation of Digital Mobility Outcomes from a Single Wearable Device
Source: Sensors (Basel). 2026 Jul 6;26(13):4294. doi: 10.3390/s26134294 (PMC13364285; doi:10.3390/s26134294)
Supplement: Supplementary file 1 [file sensors-26-04294-s001.zip › sensors-4348306-supplementary.pdf]

Supplementary material

Supplementary Tables

Supplementary text box S1. Example of the minimal code required to run the GsdIluz algorithm on the LabExampleDataset evaluation

```
from mobgap.data import LabExampleDataset
from mobgap.gait_sequences import GsdIluz, GsdIonescu
from mobgap.gait_sequences.pipeline import GsdEmulationPipeline
from mobgap.gait_sequences.evaluation import GsdEvaluation, gsd_evaluation_scorer
# Algorithm parameters are set at instantiation, separate from execution.
# Any other GSD algorithm (e.g. GsdIonescu()) could be substituted here.
algo = GsdIluz(min_bout_duration_s=3.0)
# The algorithm instance is injected into the pipeline wrapper
pipeline = GsdEmulationPipeline(algo)

# Load dataset with reference annotations
dataset = LabExampleDataset(reference_system="INDIP").get_subset(test="Test11")

# Run the evaluation challenge across all datapoints and store results
challenge = GsdEvaluation(
    dataset, scoring=gsd_evaluation_scorer
).run(pipeline)

results = challenge.results_
```

Supplementary Table S1. Selected performance measures evaluating the performance of the block-by-block performance measures. Errors obtained from the comparison between the measures provided by the INDIP system and mobgap estimated from the data provided by the single wearable device.

**Pipeline block**	**Gait sequence detection**			**Initial contact detection**			**Cadence estimation**			**Stride length estimation**		
**Cohort**	**F1 Score**	**Abs. Rel. Error [%]**	**ICC**	**Abs. Error [s]**	***Bias and LoA***	***F1 Score***	**Abs. Error [s]**	**Abs. Rel. Error [%]**	**ICC**	**Abs. Error [m]**	**Abs. Rel. Error [%]**	**ICC**
**All**	0.82 [0.79, 0.85]	17.65 [13.71, 21.59]	0.95 [0.93, 0.97]	0.06 [0.01, 0.11]	0.08 [0.03, 0.14]	0.91 [0.89, 0.93]	4.96 [4.10, 5.83]	5.89 [4.77, 7.01]	0.82 [0.74, 0.88]	0.14 [0.12, 0.15]	18.81 [16.49, 21.13]	0.71 [0.55, 0.81]
**HA (n=20)**	0.85 [0.81, 0.88]	22.78 [15.88, 29.67]	0.88 [0.03, 0.97]	0.05 [0.03, 0.08]	0.08 [0.05, 0.11]	0.93 [0.92, 0.95]	5.02 [3.82, 6.23]	5.93 [4.32, 7.54]	0.88 [0.73, 0.95]	0.10 [0.09, 0.12]	14.26 [11.33, 17.19]	0.88 [0.70, 0.95]
**COPD (n=17)**	0.76 [0.69, 0.82]	37.82 [21.48, 54.16]	0.31 [-0.13, 0.67]	0.07 [0.02, 0.12]	0.09 [0.04, 0.14]	0.91 [0.89, 0.92]	3.79 [3.05, 4.53]	4.75 [3.72, 5.77]	0.92 [0.78, 0.97]	0.15 [0.12, 0.17]	20.90 [16.38, 25.42]	0.52 [-0.06, 0.82]
**CHF (n=10)**	0.84 [0.77, 0.90]	31.12 [12.65, 49.59]	0.97 [0.55, 0.99]	0.06 [0.01, 0.10]	0.08 [0.02, 0.14]	0.94 [0.91, 0.96]	4.50 [2.92, 6.08]	4.84 [3.34, 6.34]	0.93 [0.74, 0.98]	0.12 [0.09, 0.15]	14.50 [9.61, 19.40]	0.92 [0.71, 0.98]
MS (n = 18)	0.85 [0.81, 0.89]	20.34 [13.21, 27.46]	0.94 [0.6, 0.98]	0.06 [-0.00, 0.13]	0.09 [0.0, 0.15]	0.93 [0.9, 0.95]	6.10 [3.96, 8.24]	7.23 [4.78, 9.68]	0.88 [0.7, 0.95]	0.15 [0.12,0.19]	21.44 [14.10, 28.77]	0.47 [0.0, 0.76]
PD (n = 19)	0.84 [0.78, 0.91]	20.05 [10.65, 29.46]	0.98 [0.96, 0.99]	0.06 [0.01, 0.11]	0.09 [0.04, 0.14]	0.93 [0.90, 0.96]	5.32 [1.99, 8.66]	6.45 [1.78, 11.11]	0.61 [0.22, 0.83]	0.15 [0.12, 0.18]	18.50 [13.83, 23.17]	0.60 [0.21, 0.82]
PFF (n =17)	0.72 [0.59, 0.85]	24.57 [10.94, 38.19]	0.97 [0.91, 0.99]	0.07 [0.02, 0.11]	0.08 [0.03, 0.13]	0.84 [0.73, 0.94]	4.69 [2.56, 6.81]	5.55 [3.13, 7.96]	0.82 [0.56, 0.94]	0.14 [0.11, 0.17]	22.62 [14.80, 30.45]	0.75 [0.41, 0.91]

Data presented as mean and 95% confidence intervals (CI) for each cohort.

Supplementary Table S2. Selected performance measures evaluating the performance of the block-by-block performance measures of the mobgap pipeline in comparison to the original implementation. Estimates obtained from the INDIP and the single wearable device.

**Pipeline block**	**Gait sequence detection**			**Initial contact detection**			**Cadence estimation**			**Stride length estimation**		
**Validation metric**	**F1 Score**	**Abs. Rel. Error [%]**	**ICC**	**Abs. Error [s]**	***Bias and LoA***	***F1 Score***	**Abs. Error [s/m]**	**Abs. Rel. Error [%]**	**ICC**	**Abs. Error [m]**	**Abs. Rel. Error [%]**	**ICC**
**All (mobgap)**	0.82 [0.79, 0.85]	17.65 [13.71, 21.59]	0.95 [0.93, 0.97]	0.06 [0.01, 0.11]	0.08 [0.03, 0.14]	0.91 [0.89, 0.93]	4.96 [4.10, 5.83]	5.89 [4.77, 7.01]	0.82 [0.74, 0.88]	0.14 [0.12, 0.15]	18.81 [16.49, 21.13]	0.71 [0.55, 0.81]
**All (original)**	0.81 [0.77, 0.84]	16.98 [12.83, 21.12]	0.94 [0.92, 0.96]	0.06 [0.01, 0.11]	0.08 [0.03,0.14]	0.91 [0.89, 0.93]	4.72 [3.94, 5.49]	5.28 [4.47, 6.09]	0.87 [0.81, 0.91]	0.16 [0.14, 0.18]	22.54 [18.56, 26.53]	0.53 [0.18, 0.73]
**HA (mobgap)**	0.85 [0.81, 0.88]	22.78 [15.88, 29.67]	0.88 [0.03, 0.97]	0.05 [0.03, 0.08]	0.08 [0.05, 0.11]	0.93 [0.92, 0.95]	5.02 [3.82, 6.23]	5.93 [4.32, 7.54]	0.88 [0.73, 0.95]	0.10 [0.09, 0.12]	14.26 [11.33, 17.19]	0.88 [0.70, 0.95]
**HA (original)**	0.83 [0.77, 0.88]	12.13 [4.64, 19.63]	0.93 [0.83, 0.97	0.05 [0.02, 0.08]	0.08 [0.04, 0.11]	0.93 [0.92,0.94]	4.64 [3.77, 5.51]	4.94 [4.17, 5.71]	0.88 [0.30, 0.97]	0.12 [0.10, 0.14]	15.86 [12.41, 19.31]	0.73 [0.21, 0.90]
**CHF (mobgap)**	0.84 [0.77, 0.90]	31.12 [12.65, 49.59]	0.97 [0.55, 0.99]	0.06 [0.01, 0.10]	0.08 [0.02, 0.14]	0.94 [0.91, 0.96]	4.50 [2.92, 6.08]	4.84 [3.34, 6.34]	0.93 [0.74, 0.98]	0.12 [0.09, 0.15]	14.50 [9.61, 19.40]	0.92 [0.71, 0.98]
**CHF (original)**	0.81 [0.69, 0.93]	20.25 [5.65, 34.86]	0.98 [0.91, 0.99]	0.07 [0.03, 0.12]	0.09 [0.04, 0.15]	0.90 [0.89, 0.92]	3.44 [1.69, 5.20]	3.55 [1.83, 5.27]	0.99 [0.91, 1.00]	0.13 [0.08, 0.18]	16.52 [5.88, 27.17]	0.84 [0.44, 0.96]
**COPD (mobgap)**	0.76 [0.69, 0.82]	37.82 [21.48, 54.16]	0.31 [-0.13, 0.67]	0.07 [0.02, 0.12]	0.09 [0.04, 0.14]	0.91 [0.89, 0.92]	3.79 [3.05, 4.53]	4.75 [3.72, 5.77]	0.92 [0.78, 0.97]	0.15 [0.12, 0.17]	20.90 [16.38, 25.42]	0.52 [-0.06, 0.82]
**COPD (original)**	0.76 [0.69, 0.83]	22.47 [6.94, 38.00]	0.32 [-0.20, 0.69]	0.05 [0.00, 0.10]	0.08 [0.01, 0.15]	0.94 [0.92, 0.96]	4.23 [3.45, 5.00]	4.82 [4.04, 5.60]	0.86 [0.17, 0.96]	0.18 [0.15, 0.21]	24.95 [19.88,30.02]	0.36 [-0.10, 0.74]
***MS* (mobgap)**	0.85 [0.81, 0.89]	20.34 [13.21, 27.46]	0.94 [0.68, 0.98]	0.06 [-0.00, 0.13]	0.09 [0.02, 0.15]	0.93 [0.91, 0.95]	6.10 [3.96, 8.24]	7.23 [4.78, 9.68]	0.88 [0.70, 0.95]	0.15 [0.12,0.19]	21.44 [14.10, 28.77]	0.47 [0.03, 0.76]
**MS (original)**	0.85 [0.81, 0.89]	17.22 [10.54, 23.91]	0.95 [0.79, 0.99]	0.06 [-0.00, 0.13]	0.09 [0.03, 0.15]	0.93 [0.91, 0.96]	5.33 [3.87, 6.79]	6.72 [4.45, 8.99]	0.92 [0.79,0.97]	0.18 [0.13,0.23]	27.00 [14.43, 39.58]	0.43 [-0.05, 0.75]
***PD* (mobgap)**	0.84 [0.78, 0.91]	20.05 [10.65, 29.46]	0.98 [0.96, 0.99]	0.06 [0.01, 0.11]	0.09 [0.04, 0.14]	0.93 [0.90, 0.96]	5.32 [1.99, 8.66]	6.45 [1.78, 11.11]	0.61 [0.22, 0.83]	0.15 [0.12, 0.18]	18.50 [13.83, 23.17]	0.60 [0.21, 0.82]
***PD* (original)**	0.84 [0.77, 0.90]	19.84 [10.58,	0.99 [0.96, 0.99]	0.06 [0.01, 0.10]	0.08 [0.03, 0.14]	0.93 [0.90, 0.96]	5.42 [2.48,	5.90 [2.99, 8.81]	0.70 [0.37,	0.17 [0.12, 0.23]	25.39 [11.38,	0.35 [-0.06,

		29.11]					8.36]		0.87]		39.41]	0.68]
***PFF* (mobgap)**	0.72 [0.59, 0.85]	24.57 [10.94, 38.19]	0.97 [0.91, 0.99]	0.07 [0.02, 0.11]	0.08 [0.03, 0.13]	0.84 [0.73, 0.94]	4.69 [2.56, 6.81]	5.55 [3.13, 7.96]	0.82 [0.56, 0.94]	0.14 [0.11, 0.17]	22.62 [14.80, 30.45]	0.75 [0.41, 0.91]
***PFF* (original)**	0.71 [0.58, 0.84]	24.31 [10.75, 37.86]	0.96 [0.89, 0.98]	0.06 [0.02, 0.11]	0.08 [0.03, 0.13]	0.84 [0.73, 0.95]	4.47 [1.94, 7.01]	4.70 [2.65, 6.76]	0.82 [0.51, 0.94]	0.16 [0.13, 0.19]	23.51 [16.82, 30.21]	0.48 [-0.03, 0.80]

Data presented as mean and 95% confidence intervals (CI) for each cohort.

Supplementary Table S3. Full pipeline validation across 2.5-hour real-world validation for walking speed.

Cohort	Number of WBs	Mobgap mean and CI [m/s]	INDIP mean and CI [m/s]	Bias and LoA [m/s]	Abs. Error [m/s]	Rel. Error [%]	Abs. Rel. Error [%]	ICC
**All (n=101)**	1984	0.71 [0.68, 0.73]	0.67 [0.64, 0.70]	0.04 [-0.13, 0.21]	0.10 [0.09, 0.11]*	11.84 [8.31, 15.37]	19.63 [16.94, 22.32]	0.81 [0.67, 0.88]
**CHF (n=10)**	220	0.75 [0.67, 0.84]	0.78 [0.67, 0.89]	-0.02 [-0.16, 0.12]	0.10 [0.07, 0.13]	2.72 [-4.40, 9.85]	15.32 [10.60, 20.03]	0.90 [0.67, 0.97]
**COPD (n=17)**	410	0.69 [0.66, 0.73]	0.62 [0.57, 0.66]	0.07 [-0.05, 0.20]	0.10 [0.09, 0.12]	16.51 [10.46, 22.56]	20.74 [16.55, 24.92]	0.53 [-0.08, 0.83]
**HA (n=20)**	524	0.72 [0.65, 0.78]	0.69 [0.62, 0.76]	0.02 [-0.08, 0.12]	0.08 [0.06, 0.09]	7.22 [3.70, 10.74]	13.72 [11.51, 15.93]	0.93 [0.82, 0.97]
**MS (n=18)**	327	0.77 [0.70, 0.83]	0.69 [0.63, 0.75]	0.08 [-0.10, 0.25]	0.12 [0.09, 0.15]	17.47 [9.38, 25.56]	22.69 [15.91, 29.48]	0.67 [0.12, 0.88]
**PD (n=19)**	267	0.73 [0.67, 0.79]	0.71 [0.64, 0.78]	0.02 [-0.20, 0.24]	0.11 [0.09, 0.14]	8.92 [-2.59, 20.44]	20.70 [12.08, 29.33]	0.72 [0.41, 0.88]
**PFF (n=17)**	236	0.58 [0.52, 0.63]	0.53 [0.46, 0.60]	0.04 [-0.12, 0.20]	0.10 [0.08, 0.12]	15.71 [4.68, 26.74]	24.07 [15.37, 32.77]	0.79 [0.46, 0.92]

Supplementary Table S4. Full pipeline validation across 2.5 real-world validation of the mobgap pipeline in comparison to the original implementation.

Cohort (version)	Number of WBs	WD mean and CI [m/s]	INDIP mean and CI [m/s]	Bias and LoA [m/s]	Abs. Error [m/s]	Rel. Error [%]	Abs. Rel. Error [%]	ICC
**All (mobgap)**	1984	0.71 [0.68, 0.73]	0.67 [0.64, 0.70]	0.04 [-0.13, 0.21]	0.10 [0.09, 0.11]*	11.84 [8.31, 15.37]	19.63 [16.94, 22.32]	0.81 [0.67, 0.88]
**All (original)**	1697	0.77 [0.75, 0.80]	0.71 [0.68, 0.74]	0.07 [-0.13, 0.26]	0.12 [0.11, 0.13]	17.11 [12.22, 22.01]	22.89 [18.62, 27.17]	0.69 [0.37, 0.83]
**HA (mobgap)**	220	0.72 [0.65, 0.78]	0.69 [0.62, 0.76]	0.02 [-0.08, 0.12]	0.08 [0.06, 0.09]	7.22 [3.70, 10.74]	13.72 [11.51, 15.93]	0.93 [0.82, 0.97]
**HA (original)**	410	0.79 [0.74, 0.84]	0.75 [0.69, 0.81]	0.04 [-0.09, 0.17]	0.09 [0.08, 0.11]	9.45 [4.82, 14.08]	15.36 [12.39, 18.33]	0.85 [0.59, 0.94]
**CHF (mobgap)**	220	0.75 [0.67, 0.84]	0.78 [0.67, 0.89]	-0.02 [-0.16, 0.12]	0.10 [0.07, 0.13]	2.72 [-4.40, 9.85]	15.32 [10.60, 20.03]	0.90 [0.67, 0.97]
**CHF (original)**	176	0.83 [0.72, 0.93]	0.83 [0.70, 0.96]	-0.00 [-0.19, 0.18]	0.10 [0.06, 0.14]	5.39 [-6.37, 17.15]	15.19 [5.79, 24.60]	0.89 [0.60, 0.98]
**COPD (mobgap)**	410	0.69 [0.66, 0.73]	0.62 [0.57, 0.66]	0.07 [-0.05, 0.20]	0.10 [0.09, 0.12]	16.51 [10.46, 22.56]	20.74 [16.55, 24.92]	0.53 [-0.08, 0.83]
**COPD (original)**	323	0.75 [0.71, 0.79]	0.65 [0.60, 0.69]	0.10 [-0.03, 0.23]	0.13 [0.10, 0.15]	21.58 [15.75, 27.41]	24.30 [19.58, 29.02]	0.40 [-0.11, 0.77
**MS (mobgap)**	327	0.77 [0.70, 0.83]	0.69 [0.63, 0.75]	0.08 [-0.10, 0.25]	0.12 [0.09, 0.15]	17.47 [9.38, 25.56]	22.69 [15.91, 29.48]	0.67 [0.12, 0.88]
**MS (original)**	355	0.82 [0.76, 0.88]	0.71 [0.64, 0.78]	0.11 [-0.10, 0.32]	0.15 [0.11, 0.18]	27.78 [11.34, 44.22]	31.46 [16.01, 46.90]	0.59 [-0.02, 0.85]
**PD (mobgap)**	267	0.73 [0.67, 0.79]	0.71 [0.64, 0.78]	0.02 [-0.20, 0.24]	0.11 [0.09, 0.14]	8.92 [-2.59, 20.44]	20.70 [12.08, 29.33]	0.72 [0.41, 0.88]
**PD (original)**	256	0.79 [0.74, 0.85]	0.73 [0.66, 0.81]	0.06 [-0.20, 0.32]	0.13 [0.09, 0.16]	17.07 [1.50, 32.64]	24.82 [11.17, 38.48]	0.53 [0.13, 0.78]
**PFF (mobgap)**	236	0.58 [0.52, 0.63]	0.53 [0.46, 0.60]	0.04 [-0.12, 0.20]	0.10 [0.08, 0.12]	15.71 [4.68, 26.74]	24.07 [15.37, 32.77]	0.79 [0.46, 0.92]
**PFF (original)**	177	0.66 [0.61, 0.70]	0.59 [0.54, 0.65]	0.06 [-0.11, 0.23]	0.11 [0.09, 0.14]	16.48 [7.25, 25.71]	23.29 [16.30, 30.29]	0.61 [0.09, 0.86]

Data presented as mean and 95% confidence intervals (CI) for each cohort

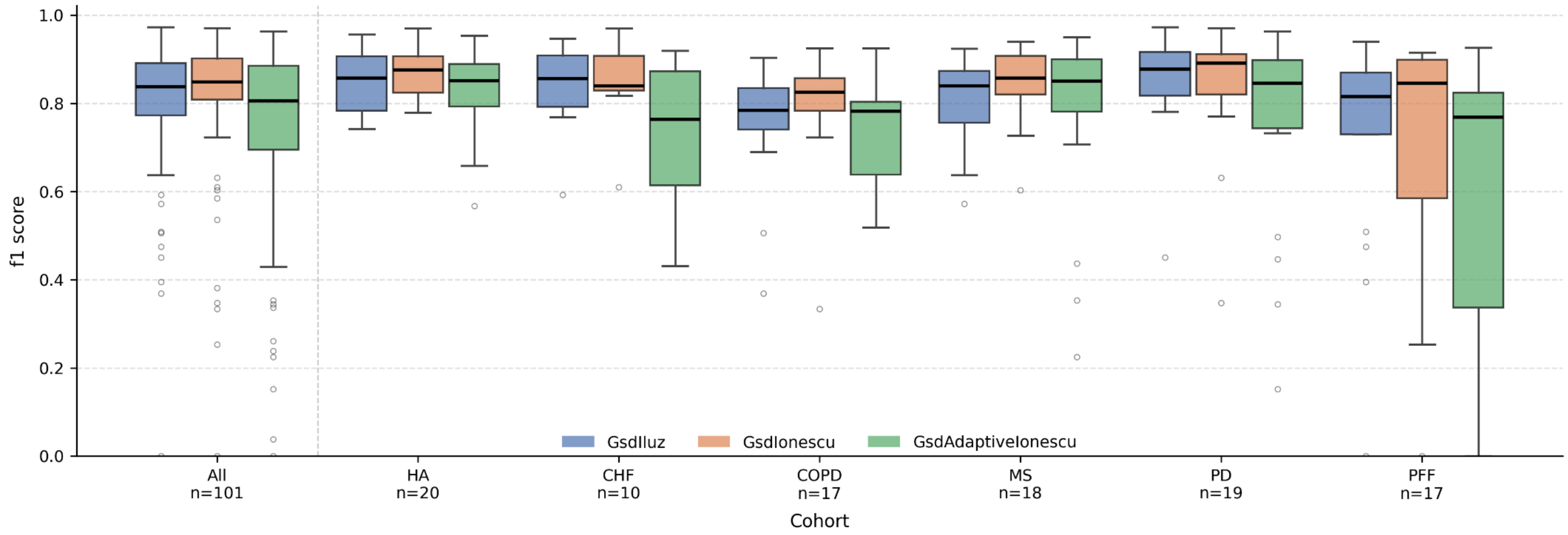

Supplementary Figure S1. Example of f1 scores compared across three gait sequence detection (GSD) algorithms that are available within mobgap repository. This figure serves as a visual representation of how any bespoke algorithm can be compared using the same standardized framework.
